# A multistage *Plasmodium* CRL4^WIG1^ ubiquitin ligase is critical for the formation of functional microtubule organization centers in microgametocytes

**DOI:** 10.1128/mbio.01672-24

**Published:** 2024-08-29

**Authors:** Ravish Rashpa, Cameron Smith, Katerina Artavanis-Tsakonas, Mathieu Brochet

**Affiliations:** 1 Department of Microbiology and Molecular Medicine, Faculty of Medicine, University of Geneva, Geneva, Switzerland; 2 Department of Pathology, University of Cambridge, Cambridge, United Kingdom; The George Washington University Milken Institute of Public Health, Washington, DC, USA

**Keywords:** *Plasmodium*, malaria, ubiquitin ligase, microtubule organization center, transmission

## Abstract

Malaria is a mosquito-borne infectious disease caused by unicellular eukaryotic parasites of the *Plasmodium* genus. Protein ubiquitination by E3 ligases is a critical post-translational modification required for various cellular processes during the lifecycle of *Plasmodium* parasites. However, little is known about the repertoire and function of these enzymes in *Plasmodium*. Here, we show that *Plasmodium* expresses a conserved cullin RING E3 ligase (CRL) complex that is functionally related to CRL4 in other eukaryotes. In *P. falciparum* asexual blood stages, a cullin-4 scaffold interacts with the RING protein RBX1, the adaptor protein DDB1, and a set of putative receptor proteins that may determine substrate specificity for ubiquitination. These receptor proteins contain WD40-repeat domains and include WD-repeat protein important for gametogenesis 1 (WIG1). This CRL4-related complex is also expressed in *P. berghei* gametocytes, with WIG1 being the only putative receptor detected in both the schizont and gametocyte stages. *WIG1* disruption leads to a complete block in microgamete formation. Proteomic analyses indicate that *WIG1* disruption alters proteostasis of ciliary proteins and components of the DNA replication machinery during gametocytogenesis. Further analysis by ultrastructure expansion microscopy (U-ExM) indicates that WIG1-dependent depletion of ciliary proteins is associated with impaired the formation of the microtubule organization centers that coordinate mitosis with axoneme formation and altered DNA replication during microgametogenesis. This work identifies a CRL4-related ubiquitin ligase in *Plasmodium* that is critical for the formation of microgametes by regulating proteostasis of ciliary and DNA replication proteins.

**IMPORTANCE:**

*Plasmodium* parasites undergo fascinating lifecycles with multiple developmental steps, converting into morphologically distinct forms in both their mammalian and mosquito hosts. Protein ubiquitination by ubiquitin ligases emerges as an important post-translational modification required to control multiple developmental stages in *Plasmodium*. Here, we identify a cullin RING E3 ubiquitin ligase (CRL) complex expressed in the replicating asexual blood stages and in the gametocyte stages that mediate transmission to the mosquito. WIG1, a putative substrate recognition protein of this ligase complex, is essential for the maturation of microgametocytes into microgametes upon ingestion by a mosquito. More specifically, WIG1 is required for proteostasis of ciliary proteins and components of the DNA replication machinery during gametocytogenesis. This requirement is linked to DNA replication and microtubule organization center formation, both critical to the development of flagellated microgametes.

## INTRODUCTION

Malaria remains a major global health problem with 249 million malaria cases and 608,000 deaths in 2022 (1). The lifecycle of *Plasmodium* parasites alternates between mosquito and vertebrate hosts. Clinical symptoms of malaria are linked to the dividing asexual blood stages, whereas transmission to the mosquito is solely initiated by sexually differentiated gametocytes. Differentiation from asexually replicating stages into gametocytes takes place inside host erythrocytes. Following a period of maturation, micro- and macrogametocytes are ready to initiate transmission when ingested by a mosquito during a blood meal (2, 3). Gametocytes resume their development in response to environmental conditions encountered in the mosquito midgut. These include a small mosquito molecule, xanthurenic acid (XA), a rise in extracellular pH, and a drop in temperature. Upon activation, gamete egress from the host erythrocyte relies on the release of secretory vesicles called osmiophilic bodies (4). The macrogamete becomes rapidly available for fertilization following activation of translationally repressed mRNAs (5, 6). By comparison, the microgametocyte undergoes a more dramatic activation process. Once in the mosquito, three rounds of genome replication followed by mitosis within an intact nuclear envelope produce eight haploid microgametes in a process called exflagellation, all in less than 15 min.

Microgametogenesis relies on the synchronization of mitosis and axoneme assembly by bipartite microtubule organization centers (MTOCs). In non-activated microgametocytes, a single bipartite MTOC lies on the cytoplasmic face of a nuclear pore that is physically linked to an intranuclear body in the nuclear face of the same pore (7). At this stage, centrin, γ-tubulin, SAS4-HA, and SAS6-HA are present in the amorphous MTOC but are not incorporated into a structure resembling known MTOC such as centrosome, basal body, or spindle pole body. Upon activation of gametogenesis, the MTOC gives rise to two tetrads of four basal bodies from the amorphous MTOC through the dynamic relocalization of SAS4, SAS6, and centrin (8). On the nuclear side, the intranuclear bodies differentiate into spindle poles that coordinate concomitant mitotic events (9). After 1–2 min, the first mitotic spindle is visible, and four basal bodies with growing axonemes that display the canonical 9 + 2 microtubule symmetry are found at each extremity of the mitotic spindle. Each of the eight axonemes polymerises in the cytoplasm in the absence of intra-flagellar transport (7, 10). After 3–5 min, mitosis II occurs during which a pair of basal bodies are found at each end of two mitotic spindles. By 6–8 min after initiation, the four spindles of mitosis III are visible and a single basal body is found attached to each of the eight spindle poles. The process of chromatin condensation begins at the end of mitosis III, when each of the eight short spindles anchors one haploid set of 14 chromosomes to its respective spindle pole. At the onset of exflagellation, the axonemes become motile and incorporate a haploid genome into the forming flagellated gamete (10).

This coordination between genome replication, closed mitosis, and axoneme assembly requires tight regulation. The role of protein phosphorylation by kinases has been shown to be crucial for the formation of microgametes (11, 12). This includes a kinase related to cyclin-dependent kinases (13), a mitogen-activated protein kinase (14, 15), an aurora kinase (16), or plant-like calcium-dependent protein kinases (17–21). More recently, ubiquitination has emerged as a critical post-translational modification in the regulation of *Plasmodium* gametogenesis. A first snapshot of the gametocyte ubiquitome revealed 2,183 ubiquitinated lysine residues mapping onto 519 proteins (22). Ubiquitination is mediated by various E3 ubiquitin ligases, grouped by their composite protein domains: HECT, U-box, PHD-finger, and RING-finger domains. So far, proteins belonging to two complexes of the RING-finger family have been shown to play important roles during microgametogenesis. The anaphase-promoting complex/cyclosome (APC/C) is a ligase that targets mitotic proteins involved in the metaphase-to-anaphase transition in other eukaryotes (23). Genome mining identified only four APC/C subunits in *Plasmodium*: a single CDC20/CDH1 homolog, APC3, APC10, and APC11 (24–26). CDC20 and APC3 were shown to be critical for exflagellation with proposed roles for mitotic spindle formation but not DNA replication nor axoneme assembly (24, 25). More recently, another multisubunit E3 ubiquitin ligase complex, the SKP1/Cullin-1/FBXO1 complex (CRL1 or SCF^FBXO1^), was shown the be crucial for multiple developmental stages during the *Plasmodium* lifecycle. This includes microgametogenesis during which, deletion or depletion of FBXO1 leads to defects in the MTOC segregation and egress from the host red blood cell (RBC) (22).

The CRL1 complex belongs to the class of cullin-RING ligases (CRLs), the largest family of ubiquitin E3 ligases with over 400 members (27). Mammalian cells express eight classes of CRL complexes, each containing at least four core components: one of eight cullin isoforms serving as a common backbone scaffold protein, one of two RING finger proteins (RBX1 or RBX2) that binds a Ub-loaded E2 ubiquitin-conjugating enzyme, a substrate receptor that recognizes the target protein, and adaptor subunits that serve as links between the cullin scaffold and substrate receptors. Cells contain a multitude of substrate receptors, which are critical for the specificity of a given CRL for its target proteins. For example, in humans, upwards of 70 F-box protein receptors have been identified for CRL1. Despite the diversity of receptor proteins, they typically display protein-protein interaction domains such as tryptophan-aspartic acid 40 (WD40), leucine-rich repeat (LRR), Src Homology 2 (SH2), or Kelch domains.

In addition to CUL1, *Plasmodium* encodes a second cullin annotated as cullin-like (PF3D7_0629800—Cullin-2 or CUL2) that shares maximum sequence identity with human Cullin-4B. PF3D7_0629800 was recently shown to be essential for intraerythrocytic proliferation and gametogenesis in *P. falciparum* and to interact with RBX1 and putative adaptors to form a CRL4-like complex (28). To keep in line with the nomenclature, we hereafter refer to this protein as CUL4.

Here, we confirm that CUL4 forms a CRL4-related complex in *P. falciparum* asexual blood stages that consists of RBX1, the adaptor protein DDB1 and a distinct set of putative receptor proteins containing WD40 repeat domains. We further show that this CRL4-related complex is also expressed and assembled in *P. berghei* gametocytes comprising a conserved WD protein named WIG1. As *P. berghei* lends itself more readily to genetic manipulation and the study of transmission stages, we explored the biology of the WD protein in this model system. While we determined that its gene disruption does not impact the proliferation of asexual blood stages, its disruption leads to a complete block in microgamete formation. We thus decided to name this protein WD repeat protein important for gametogenesis 1 (WIG1). Proteomic analyses indicate that *WIG1* disruption alters the proteostasis of ciliary proteins and components of the DNA replication machinery during gametocytogenesis. Further analysis by ultrastructure expansion microscopy (U-ExM) indicates that WIG1-dependent depletion of ciliary and DNA replication proteins is associated with altered formation of the microgametocyte MTOCs and defects in DNA replication.

## RESULTS

### CRL4-related complexes are expressed in *P. falciparum* schizonts

**Fig 1:**
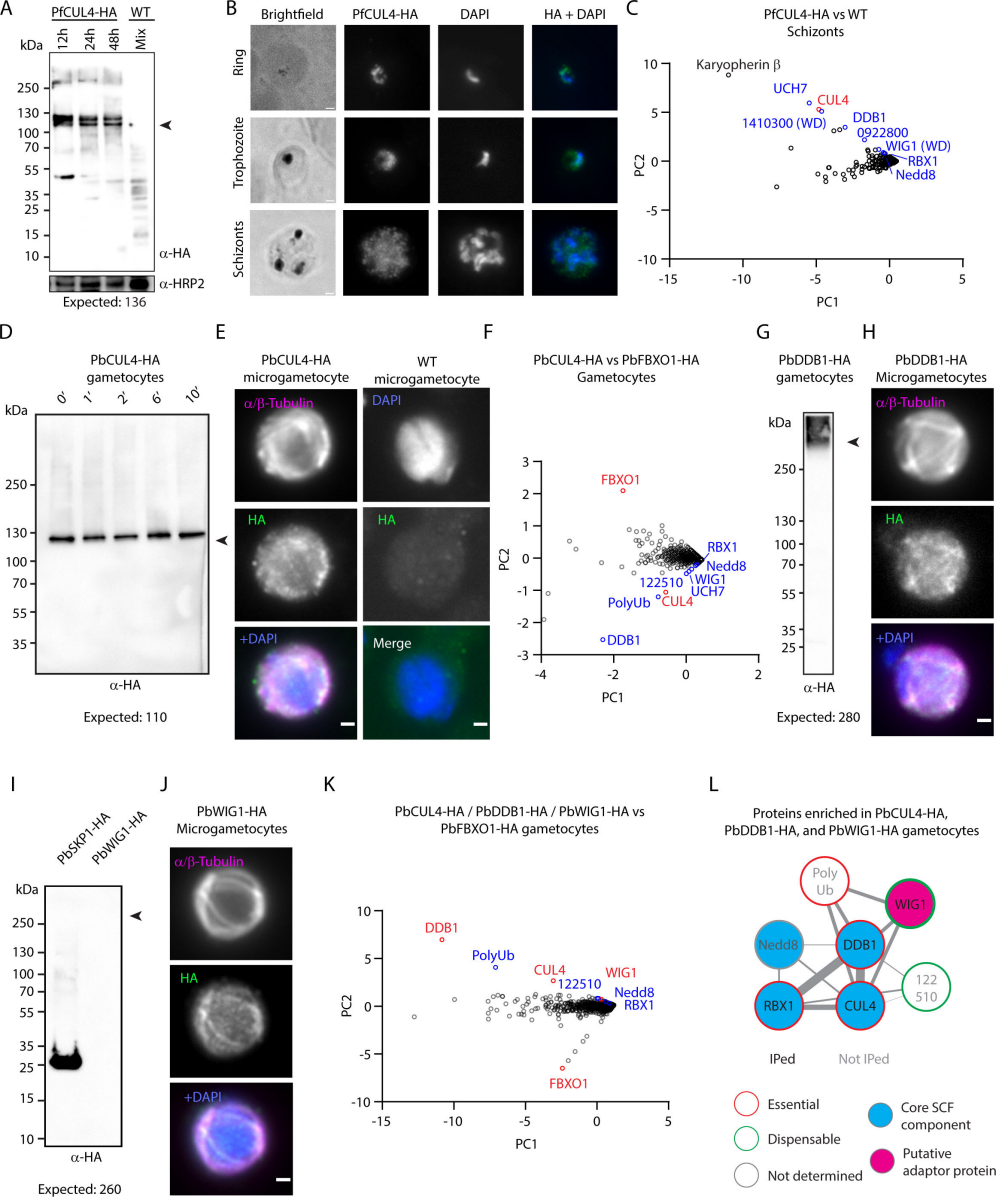
Expression of a putative CRL4^WIG1^ ubiquitin ligase complex in *P. falciparum* schizonts and *P. berghei* gametocytes. (A) Western blot analysis of PfCUL4-HA expression in *P. falciparum* PfCUL4-HA or WT lysates collected at different time points during an intraerythrocytic cycle. Staining against Histidine-Rich protein 2 (HRP2) serves as a loading control. The arrowhead indicates the expected protein size. (B) Localization of PfCUL4-HA (green) by widefield immunofluorescence in ring, trophozoite, and schizont stages. DNA is stained with DAPI (blue). Scale bar = 1 µm. (C) Two-dimensional PCA showing the enrichment of proteins identified by semiquantitative mass spectrometry in pull-downs from *P. falciparum* PfCUL4-HA compared to WT control parasites (*n* = 3). In red is the CUL4-HA bait. In blue are components of the CRL4 complex together with a protein of unknown function also identified in CRL4 IPs in reference 28, respectively. (D) Western blot analysis of PbCUL4-HA gametocyte lysates. The arrowhead indicates the expected protein size. The blot is representative of 2 independent replicates. (E) Localization of PbCUL4-HA (green) by widefield immunofluorescence in *P. berghei* gametocytes 8–10 min post-activation by XA. DNA is stained with DAPI (blue). Panels on the right show staining on a control WT microgametocyte. Scale bar = 1 µm. (F) Quantitative value (normalized total spectra) for proteins co-purifying with PbCUL4-HA and PbFBXO1-HA (control from reference 22) following immunoprecipitation, and displayed in first and second principal components (*n* = 2 biological replicates). Red denotes immunoprecipitated proteins and blue possible components of the CRL4 complex. (G) Western blot analysis of DDB1-HA expression in lysates of non-activated *P. berghei* gametocytes. The arrowhead indicates the expected protein size. (H) Localization of PbDDB1-HA by widefield immunofluorescence in *P. berghei* gametocytes 8–10 min post-activation by XA. DNA is stained with DAPI (blue). Scale bar = 1 µm. (I) Western blot analysis of a gametocyte lysate from the line expressing endogenously HA-tagged WIG1 does not allow to detect the fusion protein. The blot is representative of two independent replicates. A lane loaded with a PbSKP1-HA gametocyte lysate serves as a positive control. A Ponceau staining serving as a loading control is shown in Fig. S1E. (J) Localization of PbWIG1-HA by widefield immunofluorescence in *P. berghei* gametocytes 8–10 min post-activation by XA. DNA is stained with DAPI (blue). Scale bar = 1 µm. (K) Four-dimensional analysis of quantitative values (normalized total spectra) for proteins co-purifying with PbCUL4-HA, PbDDB1-HA, PbWIG1-HA, and PbFBXO1-HA (control) following immunoprecipitation, and displayed in first and second principal components (*n* = 2 biological replicates for each bait). Red denotes immunoprecipitated proteins and blue possible components of the CRL4 complex. (L) Protein-protein interaction network analysis of enrichment from PbCUL4-HA, PbDDB1-HA, PbWIG1-HA, and PbRBX1-HA immuno-purifications.

We first aimed to define the protein partners of Cullin-4 (PfCUL4—PF3D7_0629800) in *P. falciparum* schizonts. To do so, we generated a *P. falciparum* line expressing a HA-tagged PfCUL4 (Fig. S1A). Immunoblotting confirmed the expression of the fusion protein at all stages of intraerythrocytic development with the expected size-dependent protein mobility (Fig. 1A). A second band of heavier molecular weight was also observed. Conjugation of Nedd8 to cullins is a conserved post-translational modification universally involved in the regulation of CRL activity. As PfNedd8 is 8.6 kDa, the heavier form may correspond to neddylated PfCUL4. Immunofluorescence assays showed a cytoplasmic and nuclear distribution of PfCUL4-HA with no signal observed around hemozoin crystals suggesting its exclusion from the food vacuole (Fig. 1B).

To identify interacting proteins for PfCUL4, we affinity-purified PfCUL4-HA from synchronized transgenic *P. falciparum* schizonts followed by tandem mass spectrometry (IP-LC-MS/MS) using a WT untransfected line as a control (Fig. 1C; Table S1). Supporting the notion of a PfCUL4-based CRL complex, a two-dimensional principal component analysis (PCA) highlighted the RING finger proteins RBX1 (PF3D7_0319100) and Nedd8 (PF3D7_1313000) as enriched proteins co-purifying with PfCUL4-HA (Fig. 1C). Interestingly, one of the most enriched protein besides PfCUL4 itself was a putative orthologue of DNA damage-binding protein 1 (DDB1; PF3D7_0317700, annotated as cleavage and polyadenylation specific factor subunit A [CPSF_A]), the main adaptor of CUL4-based CRL complexes in eukaryotes. Moreover, among the proteins significantly enriched in PfCRL4-HA immunoprecipitates were also two proteins containing WD domains (PF3D7_0321800 or WIG1, and PF3D7_1410300) that could represent possible substrate receptors of the *Plasmodium* CRL4-related complex. A ubiquitin carboxyl-terminal hydrolase (PfUCH7—PF3D7_0726500), karyopherin β (PF3D7_0524000), and a protein of unknown function, PF3D7_0922800, were also significantly enriched.

### The expression of a CRL4 complex is conserved in *P. berghei* gametocytes

As CUL4 and all its identified partners share a transcription peak in sexual stages, we asked whether the CRL4-related complex was also conserved in gametocytes. To do so, we turned to the rodent malaria parasite *P. berghei* and generated a line expressing a triple HA-tagged PbCUL4 (PBANKA_1128600; Fig. S1B and C). Immunoblotting confirmed the expression of the fusion protein in mature gametocytes with a band showing the expected electrophoretic mobility (Fig. 1D; Fig. S1D). As observed in *P. falciparum* schizonts, two bands likely corresponding to non-neddylated and neddylated PbCUL4 forms were visible. No differential detection of the protein by western blot was observed during the 10 first minutes following the activation of gametocytes by XA. Immunofluorescence assays also showed a cytoplasmic and nuclear distribution of CUL4-HA both in *P. berghei* schizonts (Fig. S1E) and microgametocytes (Fig. 1E).

Affinity purification of PbCUL4-HA from 4 min activated *P. berghei* gametocytes also identified RBX1 (PBANKA_0806200), DDB1 (PBANKA_0807500), Nedd8 (PBANKA_1411400), and polyubiquitin (PolyUb; PBANKA_0610300) as enriched compared to proteins recovered from the SCF1 substrate receptor FBXO1 immuno-precipitations that was used as a control (Fig. 1F; Table S2) (22). This demonstrated the presence of a CRL4-based complex in multiple stages of the *Plasmodium* lifecycle. Interestingly, the orthologues of the WD-containing protein WIG1 (PBANKA_1216700) and the ubiquitin carboxyl-terminal hydrolase (PbUCH7—PBANKA_0210600) were also enriched. To confirm these interactions, we generated *P. berghei* lines expressing HA-tagged alleles of PbDDB1 and PbWIG1 (Fig. S1B and C). While immunoblotting and immunofluorescence assays confirmed expression of DDB1-HA (Fig. 1G and H) in gametocytes, no signal by western blotting was detected for WIG1-HA despite in-frame integration of the tag coding sequence (Fig. 1I; Fig. S1F). However, immunofluorescence assays showed a faint expression signal above background indicating a cytoplasmic and nuclear distribution of PbDDB1-HA and PbWIG1-HA in schizonts (Fig. S1E) and gametocytes (Fig. 1J). Affinity purification of PbDDB1-HA and PbWIG1-HA from 4 min activated *P. berghei* gametocytes followed by label-free semiquantitative mass spectrometry confirmed enrichment of PbCUL4, PbNedd8, PbRBX1, PbDDB1, and PbWIG1 compared with previous immunoprecipitations of the FBXO1 substrate receptor of the SCF1/CRL1 complex (22) (Fig. 1K). In addition, another conserved *Plasmodium* protein of unknown function, PBANKA_1225100, was also enriched in PbCUL4-HA and PbDDB1-HA immunoprecipitates from *P. berghei* gametocytes. Taken together, these results highlight conserved expression of a CUL4/RBX1/DDB1/WIG1 complex in both asexual (*P. falciparum* schizonts) and sexual (*P. berghei* gametocytes) stages (Fig. 1L).

### The putative CRL4 receptor WIG1 is essential for *P. berghei* microgamete formation

**Fig 2:**
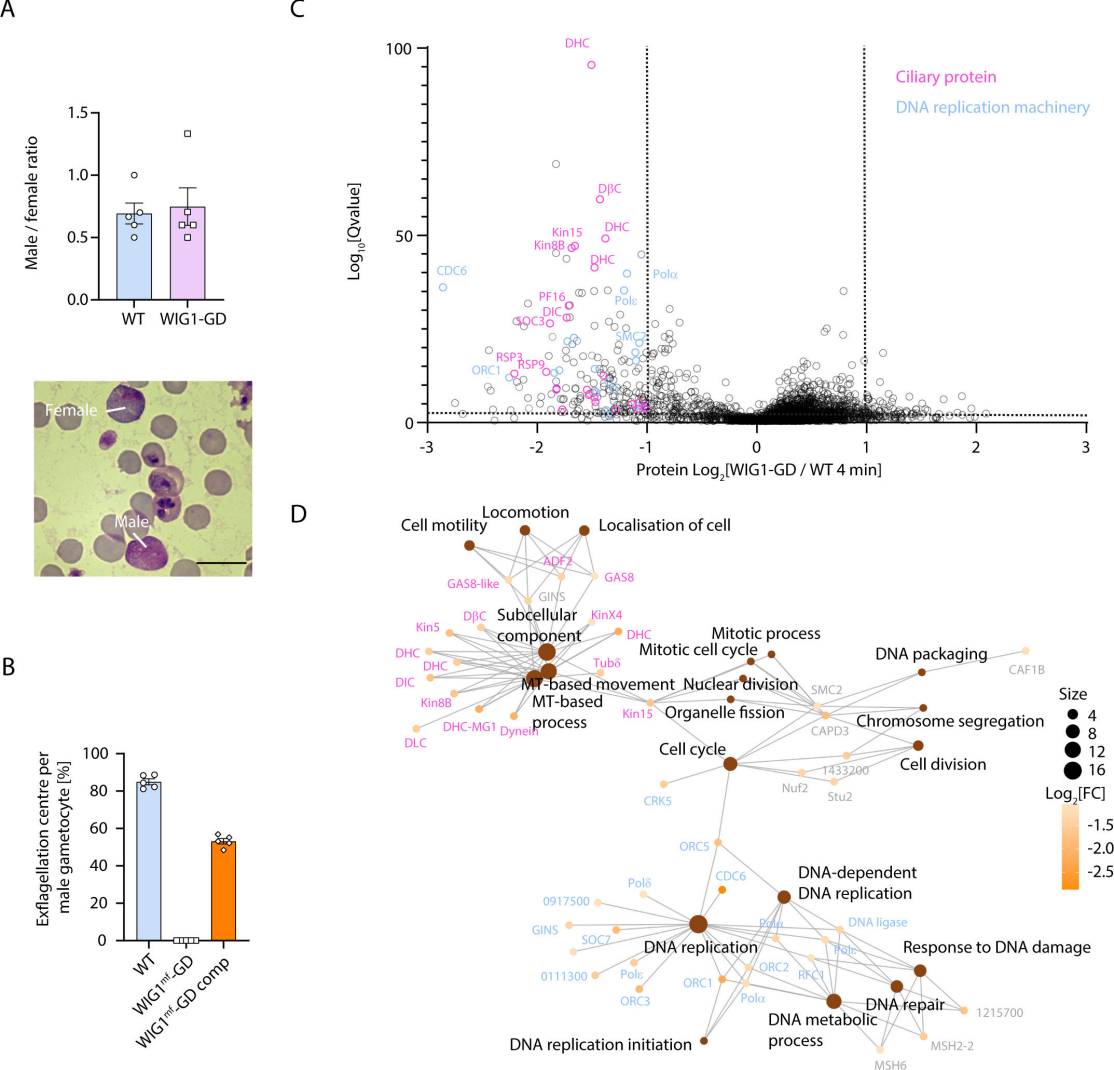
Disruption of WIG1 alters proteostasis of ciliary proteins and components of the DNA replication machinery in *P. berghei* gametocytes. (A) Disruption of *wig1* does not alter gametocyte sex ratio (*n* = 5; two-tailed *t* test). The inset shows representative examples of micro- and macrogametocytes. Scale bar = 10 µm. (B) Effect of *wig1* disruption and complementation on microgametocyte exflagellation (error bars show standard deviation from the mean; quadruplicates from three independent infections). (C) Volcano plot showing differentially detected proteins in WT and WIG1-GD gametocytes 4 min post-activation (technical duplicates from two biological replicates). (D) GO term enrichment analysis of proteins downregulated in 4 min activated WIG1-GD gametocytes.

We next set out to define the requirement of the CRL4^WIG1^ complex by focusing on WIG1. We took advantage of the highly efficient PlasmoGEM system (29, 30) to disrupt the *WIG1* gene in *P. berghei* by deleting the last 217 codons (Fig. S2A). A *WIG1* gene disruption (WIG1-GD) line was readily obtained and cloned. Quantitative PCR confirmed the absence of transcript of the deleted region, while the remaining 5′ end did not show any significant difference between the WT and transgenic lines (Fig. S2B). The WIG1-GD line produced morphologically normal macro- and microgametocytes, as assessed by Giemsa staining and microscopy (Fig. 2A). However, upon activation by XA, no exflagellation events could be observed while, on average, 82% of WT microgametocytes led to active exflagellation centers (Fig. 2B).

To confirm that the phenotype is specific to *WIG1* disruption, we used a complementation strategy. We first generated a marker-free WIG1-GD^mf^ line. By selecting for loss of the hDHFR/yCFU resistance marker with negative selection, we created a line that enabled subsequent genetic modifications (Fig. S2C). We then re-introduced at the endogenous *WIG1* locus the missing 217 codons followed by an in-frame 3×HA tag and the 3′ UTR of PbDHFR with the HA-tagging PlamsoGEM targeting vector used to generate the WIG1-HA line (Fig. S2C). Exflagellation was restored in the resulting WIG1-GD^mf^-comp line, albeit to slightly reduced levels as compared with the parental line (Fig. 2B).

### Disruption of *WIG1* alters proteostasis of ciliary proteins and components of the DNA replication machinery

We then set out to exploit the high synchronicity of developing gametocytes to identify whether the defect in exflagellation of WIG1-GD microgametocytes was linked to defective proteostasis. To do so, a data-independent acquisition (DIA)-MS method with neural network-based data processing was used to quantify the proteomes of WT and transgenic WIG1-GD gametocytes (Table S3). We focused our analysis on activated gametocytes 4 min following stimulation by XA as we previously observed a global rise in ubiquitination at this developmental stage using antibodies targeting ubiquitin (22).

Disruption of WIG1 resulted in statistically significant changes to the parasite proteome, with reduced detection of 179 proteins and increased detection of 35 proteins (*Q*-value < 10^−3^; Fig. 2C). GO term enrichment analysis indicated that proteins mapping to multiple biological processes specific to the microgametocyte were significantly downregulated in WIG1-GD gametocytes. This included microtubule-based movement, mitotic cell cycle, and DNA replication (Fig. 2D). Interestingly, proteins associated with microtubule-based movement mapped to predicted components of the axonemes that correspond to ancestral ciliary proteins (31) including eleven dynein light or heavy chains, kinesins 4X, 5, 8B, and 13, SOC3, the armadillo repeat protein PF16, and all three annotated radial spoke proteins. Proteins associated with DNA replication included ORC1 to 5, three subunits of the condensin-2 complex, various subunits of DNA polymerase α, δ, or ε and the cyclin-related kinase 5 (CRK5) that was previously shown to coordinate mitosis and DNA replication during microgametogenesis (13).

### WIG1 is required for efficient DNA replication and formation of functional MTOCs coordinating mitotic spindle and axoneme assembly

**Fig 3:**
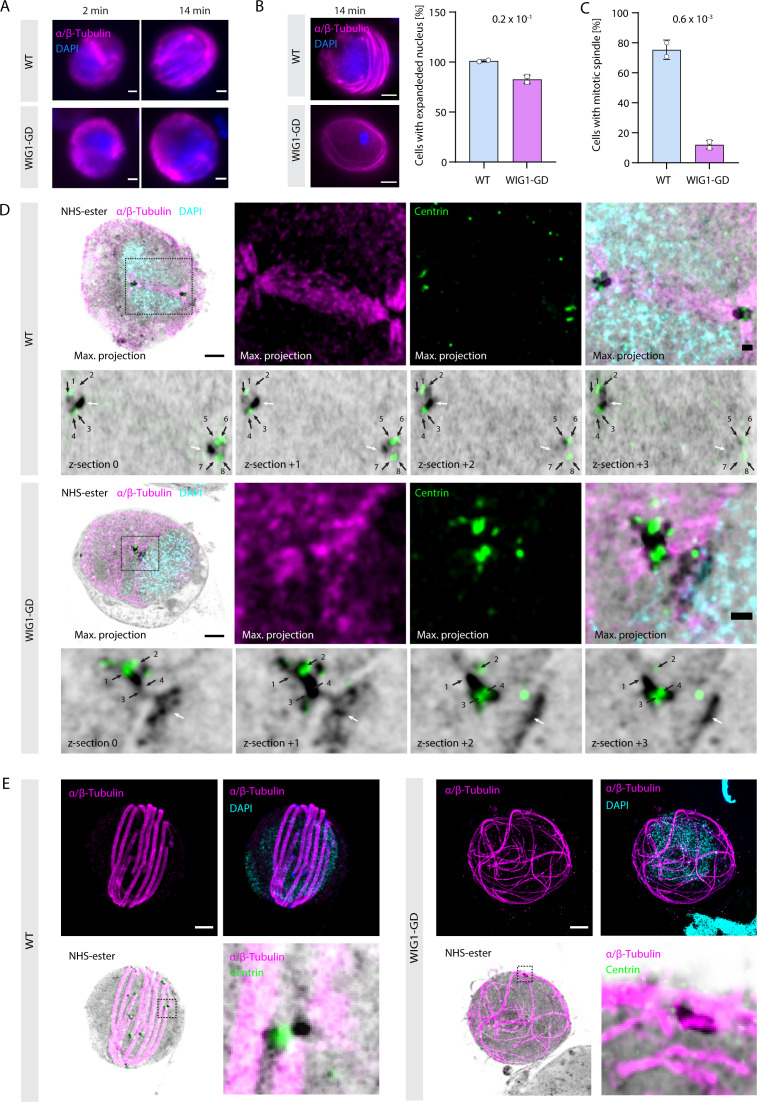
WIG1 is critical for axoneme bundling, mitotic spindle formation, and efficient DNA replication during *P. berghei* microgametogenesis. (A) Widefield images of WT and WIG1-GD microgametocytes 2 and 14 min post-activation by XA. α/β-Tubulin are shown in magenta and DAPI-stained DNA in blue. Scale bars = 1 µm. (B) Analysis of DNA replication by U-ExM in *P. berghei* gametocytes. Images on the left are representative of an expanded nucleus (top) or non-expanded nucleus (bottom). α/β-Tubulin are shown in magenta and DAPI-stained DNA in blue. Scale bars = 5 µm; 148 WT and 169 WIG1-GD gametocytes were analyzed from two biological replicates; error bars show standard deviation from the mean; two-tailed *t* test. (C) Analysis of mitotic spindle formation by U-ExM as shown in panel D in *P. berghei* gametocytes. Twenty-five WT and 30 WIG1-GD gametocytes from two biological replicates were analyzed, error bars show standard deviation from the mean; two-tailed *t* test. (D) Representative U-ExM images of WT and WIG1-GD gametocytes 1 min post-activation by XA. For each line, the top left images are maximum projections of entire cells. Adjacent to each are details of the mitotic spindle (WT) or the related area (WIG1-GD) corresponding to the boxed areas. Below are four confocal sections detailing the MTOC. White arrows: intranuclear body or spindle pole; black arrows: centrin-positive basal bodies. Centrin: green; protein density as assessed by NHS-ester: shades of grey; α/β-tubulin: magenta; DNA stained with DAPI: cyan. Scale bars, entire cells = 5 µm; scale bars, insets = 1 µm. (E) Representative U-ExM maximum projections of WT and WIG1-GD gametocytes 10 min post-activation by XA. Bottom right panels show details of the MTOC corresponding to the boxed areas. centrin: green; protein density as assessed by NHS-ester: shades of grey; α/β-tubulin: magenta; DNA stained with DAPI: cyan. Scale bars = 5 µm.

In light of the differences observed in the WIG1-GD line at the proteome level, we decided to further refine the cellular phenotype of transgenic parasites during microgametogenesis in comparison with WT parasites. Preliminary observations by conventional immunofluorescence microscopy with DNA and α/β-tubulin staining on 2 and 14 min activated microgametocytes (Fig. 3A and B) suggested a slight defect in DNA replication as demonstrated by the absence of expanded DNA staining in a limited subset of cells upon activation. In addition, mitotic spindles were not observed in WIG1-GD gametocytes 2 min post-activation in stark contrast with WT cells. Finally, the signal corresponding to axonemes encircling the DAPI staining was less marked in WIG1-GD gametocytes 14 min post-activation suggesting defects in axoneme assembly.

To further investigate potential defects during microgametogenesis, we compared mutant and WT gametocytes using U-ExM (8). The extent of DAPI staining allowed us to identify microgametocytes that replicated DNA, at least partially, as shown in Fig. 3B. While most WT microgametocytes showed a large DAPI stained nucleus, 19% of WIG1-GD microgametocytes displayed a condensed DAPI signal suggestive of impaired DNA replication in these cells (32). A relative reduction in DNA content for cells initiating DNA replication was further confirmed by flow cytometry with a twofold accumulation of 2N cells and a fourfold reduction in 4N and 8N cells in WIG1-GD microgametocytes (Fig. S3A). Co-staining of 1 min activated microgametocytes with α/β-tubulin, centrin-1, and NHS-ester linked the absence of mitotic spindles to a defect in the replication of the bipartite MTOC (Fig. 3C and D). WT microgametocytes displayed two tetrads of four basal bodies at each extremity of the mitotic spindle highlighted by the localization of centrin dots, as a marker of the inner core of the basal bodies (8), localizing in NHS-ester-dense MTOC. However, in activated WIG1-GD microgametocytes, 80% of observed cells displayed a single NHS-ester dense MTOC with only 15% of cells containing a discernible mitotic spindle. In addition, 58% of WIG1-GD microgametocytes lacked well-defined centrin dots at the base of growing microtubule, suggesting defects in the *de novo* assembly of the cytoplasmic basal bodies and possibly of the intranuclear mitotic MTOC (Fig. 3D; Fig. S3B and C). In 10-min activated WIG1-GD microgametocytes, NHS-ester, and centrin staining revealed the absence of mitotic spindles and only a single bipartite MTOC per cell could be distinguished. Interestingly, while microtubules stained by α/β-tubulin antibodies nucleated from this single MTOC, they rarely assembled into bundled axonemes as observed in WT microgametocytes (Fig. 3E; Fig. S3B and C).

## DISCUSSION

We have identified and characterized a cullin ring ligase complex that is expressed at multiple stages of the *Plasmodium* lifecycle including asexual blood stages and microgametocytes. This complex is related to the eukaryotic cullin-4-based CRL4 ligases, which have been shown to mediate various processes including DNA repair, chromatin remodeling, and cell cycle progression in metazoans, yeast, and plants (33). *CUL4* is present as a single gene in *Schizosaccharomyces pombe*, *Caenorhabditis elegans*, *Drosophila*, and *Arabidopsis*, whereas mammalian cells express two closely related paralogues, *CUL4A* and *CUL4B*. Here, we show that *Plasmodium* CUL4 interacts with RBX1 and DDB1. Through DDB1, these complexes can associate with numerous DDB1- and CUL4-associated factors (DCAF), which directly interact with specific targets promoting their ubiquitination and subsequent degradation by the proteasome. A characteristic feature of the majority of DCAF proteins that associate with DDB1 is the presence of a WD motif. The WD repeat (also known as WD40 or beta-transducin repeat) is a short motif of approximately 40 amino acids frequently terminating with a tryptophan-aspartic acid dipeptide. WD-repeat proteins coordinate multi-protein complex assemblies, where the repeating units serve as a rigid scaffold while sequences outside the repeats themselves determine the specificity of the interaction. Immunoprecipitation of CUL4-HA in *P. falciparum* schizonts identified three WD-repeat proteins with a significant enrichment for WIG1 and PF3D7_1410300, while immunoprecipitations of CUL4-HA or DDB1-HA only identified one WD-containing protein, WIG1, in *P. berghei* gametocytes. Genome mining previously identified 80 genes coding for WD40 repeat-containing proteins in *Plasmodium* (34) and it is thus possible that many other WD proteins may serve as substrate receptors at different stages of the *Plasmodium* lifecycle.

Depletion of CUL4 in *P. falciparum* strongly impaired intraerythrocytic development of both asexual and sexual stages (28). This was associated with the deregulation of various pathways at the transcriptome level likely reflecting a pleiotropic requirement for CRL4-based complexes in *Plasmodium*. As depletion of CUL4 likely affects all CRL4s, we wondered whether targeting a single putative substrate receptor would allow us to dissect requirements for *Plasmodium* CRL4s in more detail. We turned our attention to WIG1 that was shown to interact with CUL4 both in *P. falciparum* schizonts and *P. berghei* gametocytes. To better understand the role of CUL4^WIG1^, we generated a *P. berghei* transgenic clone in which *WIG1* was disrupted. This genetic modification did not affect the growth of asexual blood stages suggesting that WIG1 is either not required during these stages or that other WD repeat proteins may complement WIG1 disruption. However, *WIG1* disruption completely abolished the formation of microgametes. This defect is likely due to the altered *de novo* formation of the bipartite MTOCs that coordinate mitosis with axoneme formation during microgametogenesis. This leads to strong defects in the formation of mitotic spindles in the nucleus and the defective axoneme bundling despite the nucleation of axonemal microtubules.

Defects observed during microgametogenesis are likely the consequence of altered proteostasis during gametocytogenesis as *WIG1* disruption is associated with downregulation of a large set of ciliary proteins. This includes dynein heavy and light chains, kinesins, and radial spoke proteins. Interestingly, previous work using expansion and electron microscopy showed that Kinesin-8B or radial spoke protein 9 (RSP9) are not required for microtubule nucleation but are necessary for the 9 + 2 symmetry of axoneme formation and hence, production of viable microgametes (35–38). However, these mutations did not affect mitotic spindle formation suggesting that the observed WIG1-GD phenotype is not solely linked to depletion of Kin-8B or RSP9. Interestingly, deletion of the gene coding for the serine/arginine-rich splicing factor protein kinase-1 (SRPK1; PBANKA_0401100) was linked with misincorporation of centrin in the amorphous MTOC leading to similar defects in axoneme and mitotic spindle assembly (8). Although not enriched, peptides of SRPK1 were identified in PfCUL4-HA and PbCUL4-HA raising the possibility of a functional link between SRPK1 and CRL4^WIG1^. In addition to ciliary proteins, another large set of components of the DNA replication machinery was downregulated including various subunits of DNA polymerases and most proteins of the origin recognition complex. These changes were also associated with impaired DNA replication defects in at least 19% of observed cells, as assessed by immunofluorescence assays. However, the exact molecular links between CRL4^WIG1^-dependent ubiquitination and the observed phenotypes remain unknown and more work focusing on ubiquitination during gametocytogenesis will be required to identify the direct targets of CRL4^WIG1^ that lead to defective microgametogenesis. As disruption of WIG1 seems to affect microgametocytogenesis, it remains unknown whether the protein is active and required during gametogenesis or during later lifecycle stages. Further studies will be required to determine the respective role of ubiquitination by APC/C, CRL1/SCF^FBXO1^, or CRL4 ubiquitin ligases at these developmental stages.

Altogether, we show that *Plasmodium* parasites express a CRL4-like complex both in the schizont and gametocyte stages. A putative CRL4 substrate receptor, WIG1, is expressed at both stages. While its disruption does not affect asexual blood stages, it impacts DNA replication and *de novo* formation of the MTOCs that coordinate mitotic spindle assembly and axoneme biogenesis during microgametogenesis. In addition to the previously described requirement of at least two other ubiquitin E3 ligases, the APC/C (24, 25) and SCF^FBXO1^ (22) complexes, this work further highlights the key role of ubiquitin ligases to the formation of microgametes which are essential for malaria transmission. CRLs are emerging as promising drug targets against different types of cancer (39) and other chronic and infectious diseases (40). The work presented here extends this potential to malaria and highlights the CRL4 complex as a potential target to block *Plasmodium* transmission.

## MATERIALS AND METHODS

### *P. falciparum* maintenance and transfection

*P. falciparum* strain NF54 was cultured as described in reference 41. Briefly, parasites were cultured within O+ human erythrocytes at 5% hematocrit in RPMI 1640 complete medium containing l-glutamine and 25 mM HEPES (Sigma), supplemented with 0.2% sodium bicarbonate, 0.5% Albumax II (Gibco), 0.36 mM hypoxanthine (Sigma), and 50 mg/L gentamicin sulfate (Melford) under a 90% N_2_/5% O_2_/5% CO_2_ gaseous atmosphere at 37°C, within a hypoxic incubator chamber (Billups-Rothenburg). Parasites were maintained at a parasitemia of 0.2–10%, calculated by counting the percentage of parasitized erythrocytes of Hemacolor (Sigma) stained thin blood smears using light microscopy. A two-stage process of Percoll separation and selective sorbitol lysis was used to synchronize *Plasmodium* within a 2-h window. Percoll is a density separation reagent, and sorbitol is an osmotic lysis agent that does not affect ring-stage parasites. A 65% Percoll solution was prepared with phosphate-buffered saline (PBS). *Plasmodium*-infected erythrocyte culture was centrifuged at 800 × *g* for 5 min and the culture supernatant discarded. The cell pellet was resuspended to 10% hematocrit and applied slowly atop the Percoll solution. The volume of the Percoll solution was proportional to the volume of culture to be applied on top (i.e., an 8-mL solution was used to separate a 10-mL 5% hematocrit culture). The layered suspension was centrifuged at 1,500 × *g* for 10 min without brake; the parasites migrated to the interface between the 65% Percoll and their culture medium while uninfected RBCs and ring-stage-infected RBCs were pelleted. The parasites at the interface were recovered and transferred to a new vessel and washed two times in incomplete media before resuspension to 5% hematocrit in complete media. This suspension was agitated at 37°C for 2 h to facilitate merozoite infection. Following incubation, the culture was pelleted and resuspended in 5% sorbitol (in PBS) for 10 min prior to two washes in incomplete media before the infected blood was resuspended and cultured as normal.

Infected erythrocytes at a parasitemia of 8–10% were collected by centrifugation at 800 × *g* for 10 min. The supernatant was aspirated, and the pellet was washed in 10 volumes of PBS, followed by centrifugation at 800 × *g* for 10 min at 4°C. A volume of 0.2% (wt/vol) saponin equivalent to the starting culture was used to resuspend the cell pellet. This suspension was incubated for 10 min at 4°C. Following centrifugation at 3,200 × *g*, the supernatant was removed, and the parasite pellet was washed three times in ice-cold PBS. Following the final wash, the supernatant was aspirated, and the pellet was flash-frozen in liquid nitrogen and stored at −80°C until use.

For transfection, infected human erythrocytes at 10% ring-stage parasitemia were pelleted by centrifugation at 800 × *g* for 5 min. The media were aspirated, and 100 µL of the erythrocyte pellet was resuspended in warm 300 µL cytomix (120 mM KCl, 0.15 mM CaCl_2_, 2 mM EGTA, 5 mM MgCl_2_, 10 mM K_2_HPO_4_/KH_2_PO_4_, and 25 mM HEPES, pH 7.6) containing 100 µg plasmid DNA. The suspension was electroporated (310 V, 950 µF, 200 Ω, exponential) in a 2-mm electroporation cuvette with a GenePulser II (Bio-Rad). Electroporated parasites were immediately transferred into 5 mL complete culture containing 100 µL fresh, washed erythrocytes (50% hematocrit). After 2 h, the complete media were replaced; after 6 more hours selection compound was added at an appropriate concentration. Upon resurgence of drug-resistant parasites, cultures were maintained under selection.

*Plasmodium* parasites transfected with the pSLI plasmid (42) containing a 1 kb homology region, were treated with 5 nM WR99210 6 h after transfection. Every day for 5 days, the media and WR99210 were refreshed; after this, the media change occurred every second day. Following resurgence, parasites were expanded, and the continuing culture was subject to selection with 0.4 mg/mL G418 in complete media. Following resurgence after this selection, parasites were expanded; those parasites being maintained in culture were subjected to double selections at the above concentrations during continuous culture.

### *P. berghei* maintenance and transfection

*P. berghei* ANKA strain clone 2.34 together with derived transgenic lines, were grown and maintained in CD1 outbred mice. Six- to twelve-week-old mice were obtained from Charles River Laboratories, and females were used for all experiments. Mice were specifically pathogen free (including *Mycoplasma pulmonis*) and subjected to regular pathogen monitoring by sentinel screening. They were housed in individually ventilated cages furnished with a cardboard mouse house, tunnel, and Nestlet, maintained at 21 ± 2°C under a 12-h light/dark cycle, and given commercially prepared autoclaved dry rodent diet and water *ad libitum*. The parasitemia of infected animals was determined by microscopy of methanol-fixed and Giemsa-stained thin blood smears.

For gametocyte production, parasites were grown in mice that had been phenyl hydrazine-treated 3 days before infection. One day after infection, sulfadiazine (20 mg/L) was added to the drinking water to eliminate asexually replicating parasites. Microgametocyte exflagellation was measured 3 or 4 days after infection by adding 4 µL of blood from a superficial tail vein to 70 µL exflagellation medium (RPMI 1640 containing 25 mM HEPES, 4 mM sodium bicarbonate, 5% fetal calf serum [FCS], and 100 µM xanthurenic acid, pH 7.4) in duplicates at least. An exflagellation event was defined as moving flagellated parasites forming clumps (exflagellation centers) with nearby red blood cells. To calculate the number of exflagellation centers per 100 microgametocytes, the percentage of RBCs infected with microgametocytes was assessed on Giemsa-stained smears. For gametocyte purification, parasites were harvested in suspended animation medium (SA; RPMI 1640 containing 25 mM HEPES, 5% FCS, and 4 mM sodium bicarbonate, pH 7.20) and separated from uninfected erythrocytes on a Histodenz cushion made from 48% of a Histodenz stock (27.6% [wt/vol] Histodenz [Sigma/Alere Technologies] in 5.0 mM Tris HCl, 3.0 mM KCl, and 0.3 mM EDTA, pH 7.20) and 52% SA, final pH 7.2. Gametocytes were harvested from the interface. To induce degradation of AID/H-tagged proteins, 1 mM auxin dissolved in ethanol (0.2% final concentration) was added to purified gametocytes for 1 h prior to activation by XA.

Transfections of *P. berghei* parasites were performed as previously described (43) with some modifications. Schizonts for transfection were purified from overnight *in vitro* culture on a Histodenz cushion made from 55% of the Histodenz stock and 45% PBS. Parasites were harvested from the interface and collected by centrifugation at 500 × *g* for 3 min, resuspended in 25 µL Basic Parasite Nucleofector solution (Lonza) and added to 10–20 µg DNA dissolved in 10 µL H_2_O. Cells were electroporated using the FI-115 program of the Amaxa Nucleofector 4D. Transfected parasites were resuspended in 200 µL fresh RBCs and injected intraperitoneally into mice. Parasite selection with 0.07 mg/mL pyrimethamine (Sigma) in the drinking water (pH ~4.5) was initiated 1 day after infection. Each mutant parasite was genotyped on a single genomic DNA preparation by PCR using three combinations of primers, specific for either the WT or the modified locus on both sides of the targeted region (experimental designs are shown in Fig. S1 and S2). For allelic replacements, sequences were confirmed by Sanger sequencing using the indicated primers. Parasite lines were cloned when indicated.

### Generation of DNA targeting constructs

The oligonucleotides used to generate and genotype the mutant parasite lines are in Table S4 and a summary of background and generated parasite lines can be found in Table S5. Triple HA and KO targeting vectors were generated using phage recombineering in *Escherichia coli* TSA strain with PlasmoGEM vectors (https://plasmogem.umu.se/pbgem/) using sequential recombineering and gateway steps as previously described (29, 44). For each gene of interest (*goi*), the zeocin resistance/Phe sensitivity cassette was introduced using oligonucleotides *goi* HA-F x *goi* HA-R and *goi* GD-F x *goi GD*-R for 3×HA and GD targeting vectors, respectively. Insertion of the GW cassette following gateway reaction was confirmed using primer pairs GW1 x *goi* QCR1 and GW2 x *goi* QCR2. The modified library inserts were then released from the plasmid backbone using NotI. The GD and triple HA targeting vectors were transfected into the 2.34 line. Schematic representations of the targeting constructs as well as WT and recombined loci are shown in Fig. S1 and S2.

### Immunofluorescence assays

*P. berghei* immunofluorescence assays were performed as described in reference 8. Four percent formaldehyde fixed cells were sedimented onto poly-d-lysine coated microscopy glass slides and briefly incubated with 0.2% Triton X-100 for 5 min and blocked with 3% bovine serum albumin for 1 h. After primary and secondary antibody incubations and washings, slides were then left to dry and cells were immediately mounted in Vectashield antifade mounting media with DAPI. Images were acquired on a widefield microscope Zeiss Axio Imager M2 using 100× objective.

For *P. falciparum* IFAs, 1 mL of culture was pelleted by centrifugation, and washed once with PBS. The cell pellet was resuspended in 4% formaldehyde (in PBS) and fixed for 1 h at room temperature with rotation. Fixed cells were pelleted and resuspended in 1 mL 0.1% Triton X-100 (in PBS) for 10 min with rotation. Permeabilized cells were pelleted and resuspended in 1% BSA (in PBS) for 1 h with rotation. A 1:1,000 dilution of primary antibody (anti-HA clone 3F10 high affinity, Roche) in 1% BSA was incubated with the cell suspension for 1 h at room temperature with rotation. Cells were pelleted and washed three times with PBS prior to resuspension in a 1:5,000 dilution of secondary antibody (goat anti-rat-Alexa Fluor 488, Thermo Fisher) in 1% BSA. Cells were incubated with rotation for 1 h at room temperature with rotation. Cells were pelleted and washed two times with PBS, then incubated with 1 µg/mL DAPI (in PBS) for 10 minutes. The cells were washed twice with distilled water, before being resuspended to 50% hematocrit in distilled water. A thin smear was prepared on poly-l-lysine coated microscopy slides and left to air dry before mounting in ProLong Diamond antifade mountant (Thermo Fisher). Images were captured using a fluorescence widefield microscope and processed using Fiji/ImageJ.

### Expansion microscopy

U-ExM was performed as described in references 8, 38. Formaldehyde fixed samples were settled on a 12-mm round poly-d-lysine (A3890401, Gibco) coated coverslips for 10–12 min. Coverslips were incubated for 5 h in 1.4% formaldehyde (FA)/2% acrylamide (AA) at 37°C. Thereafter gelation was performed in ammonium persulfate (APS)/Temed (10% each)/monomer solution (23% sodium acrylate; 10% AA; 0.1% BIS-AA in PBS) for 1 h at 37°C. Gels were denatured for 1 h and 30 min at 95°C. After denaturation, gels were incubated in distilled water overnight for complete expansion. The following day, gels were washed in PBS two times for 15 min to remove excess of water. Gels were then incubated with primary antibodies at 37°C for 3 h, and washed three times for 10 min in PBS-Tween 0.1%. Incubation with secondary antibodies was performed for 3 h at 37°C, followed by three washes of 10 min each in PBS-Tween 0.1% (all antibody incubation steps were performed with 120–160 rpm shaking at 37°C). Directly after antibody staining, gels were incubated in 1 mL of 594 NHS-ester (Merck: 08741) diluted at 10 µg/mL) in PBS for 1 h and 30 min at room temperature on a shaker. The gels were then washed three times for 15 min with PBS-Tween 0.1% and expanded overnight in ultrapure water. Gel pieces of 1 cm × 1 cm were cut from the expanded gel and attached on 24 mm round poly-d-lysine (A3890401, Gibco) coated coverslips to prevent gel from sliding and to avoid drifting while imaging. The coverslip was then mounted on a metallic O-ring 35 mm imaging chamber (Okolab, RA-35-18 2000-06) and imaged. Images were acquired on Leica TCS SP8 microscope with HC PL Apo 100×/1.40 oil immersion objective in lightning mode to generate deconvolved images. System optimized z stacks were captured between frames using HyD as detector. Images were processed with ImageJ and LAS X softwares. A list of used antibodies and dyes can be found in Table S5.

### Quantitative real-time PCR

Total RNA was isolated from purified gametocytes using the RNA purification kit from Qiagen, RNeasy Mini kit (74104). During RNA purification steps, RNase-Free DNAase set Qiagen (79254) was used to remove any DNA contamination. cDNA synthesis was performed using the GoScript Reverse Transcription system from Promega, 1 µg of purified RNA was used for each reaction. Gene expression was quantified using a KAPA SYBR FAST qPCR Master Mix (2×) Kit (Merck). For qPCRs, 100 nM primer concentration was used and analysis was conducted using the Bio-Rad CFX96 system. Three replicates were performed for each assayed gene. The histone H3 (PBANKA_0108800) and alpha-tubulin 2 (PBANKA_0522700) encoding genes were used as internal control reference genes.

### Flow cytometry analysis of gametocyte DNA content

DNA content of microgametocytes was determined by flow cytometry measurement of fluorescence intensity of purified gametocytes stained with Vybrant dye cycle violet (V35003, Life Technologies) as previously described (13, 20). Gametocytes were purified and resuspended in 100 µL of SA. Activation was induced by adding 100 µL of exflagellation medium (RPMI 1640 containing 25 mM HEPES, 4 mM sodium bicarbonate, 5% FCS, and 200 µM xanthurenic acid, pH 7.8). To rapidly block gametogenesis, 1 mL of ice-cold PBS was added and cells were stained for 20 min at 4°C with Vybrant dye cycle violet and analyzed. Gating was performed to exclude RBCs and schizont contamination from gametocytes and ploidy was analyzed using BD FACSDiva 8.0.2 software. Per sample, >50,000 cells were analyzed.

### Immunoprecipitations

#### Sample preparation for proteomic characterization of *P. falciparum* schizonts

For PfCUL4-HA immunoprecipitations, 490 mL late-stage schizont culture at 8% parasitemia was grown and harvested per sample. Frozen parasite pellets were thawed on ice, before being resuspended in ice-cold, sterile filtered lysis buffer (50 mM HEPES [pH 7.5], 150 mM NaCl, 1% Triton X-100, Complete Mini protease inhibitor). The cell suspension was lysed by five freeze-thaw cycles with agitation and vortex mixing between each cycle. The insoluble fraction was pelleted by centrifugation at 17,000 × *g* for 30 min at 4°C, and the soluble fraction was aspirated. Protein concentration in the soluble fraction was estimated using Pierce BCA protein assay kit (Thermo Fisher) following the manufacturer’s instructions. Total protein concentration was normalized to 10 mg input across samples. Twenty-five microliters pre-washed magnetic anti-HA resin (Thermo Fisher) was added to each lysate sample and incubated for 2 h at 4°C with rotation. Beads were gathered by magnet, the supernatant aspirated, and the beads washed three times (50 mM HEPES [pH 7.5], 150 mM NaCl, and 0.1% Triton X-100, Complete Mini protease inhibitor) with a change of vessel between the second and third washes. The wash solution was aspirated, and the beads were resuspended in 25 µL of 1 mg/mL HA peptide (in 15 mM Tris [pH 7.4], 150 mM NaCl, 0.1% SDS, and 0.5% NP40) and incubated for 30 min at 37°C with agitation. The eluant was collected and introduced into an SDS-PAGE gel for 5 min. The gel was fixed (as above) and transferred to the mass spectrometry facility for processing and LC-MS/MS (Department of Biochemistry, University of Cambridge).

For analysis of the HA-mediated immunoprecipitation of PfCUL4-HA, raw data were provided as output from the mass spectrometry facility at the University of Cambridge. This was used as input for MaxQuant v.2.0.3.1. Parameters were set as default with exceptions: deamidation (NQ) and GlyGly (K) were included as variable modifications; label min ratio count was set to 1 and deamidation was included as a modification used in protein quantification, while discard unmodified counterpart peptides was unchecked; the FTMS MS/MS was set to 0.05 Da and the ITMS MS/MS match tolerance was set to 0.6 Da; and iBAQ LFQ was checked. The PlasmoDB v61 proteome fasta file was used as the reference proteome file. The resultant ProteinGroup output file was input into Perseus v1.6.15.0 for further analysis using the iBAQ LFQ values. Column values were filtered based on categorical columns: only identified by site, reverse, and potential contaminant. Samples were grouped as control or experimental by categorical row annotation. iBAQ LFQ values were transformed by log2(*x*), and invalid values were filtered with a minimum requirement of 2 valid values per group in the case triplicates. Invalid values were imputed from a normal distribution: width (0.3), downshift (1.8). As a triplicate experiment, a volcano plot was generated using 250 randomizations, FDR 0.05, and S0 0.1; enriched proteins positioned above the *P* < 0.05 threshold were taken as hits.

#### Sample preparation for proteomic characterisation of *P. berghei* gametocyte immunoprecipitates

Co-immunoprecipitations (IPs) of proteins were performed with purified gametocytes from two or three independent biological replicates, as previously described (13, 22, 45). Samples were fixed for 10 min with 1% formaldehyde. Parasites were lysed in RIPA buffer (50 mM Tris HCl [pH 8], 150 mM NaCl, 1% NP-40, 0.5% sodium deoxycholate, and 0.1% SDS) and the supernatant was subjected to affinity purification with anti-HA antibody (Sigma) conjugated to magnetic beads. Beads were re-suspended in 100 µL of 6 M urea in 50 mM ammonium bicarbonate (AB). Two microliters of 50 mM dithioerythritol (DTE) was added and the reduction was carried out at 37°C for 1 h. Alkylation was performed by adding 2 µL of 400 mM iodoacetamide for 1 h at room temperature in the dark. Urea was reduced to 1 M by the addition of 500 µL AB and overnight digestion was performed at 37°C with 5 µL of freshly prepared 0.2 µg/µL trypsin (Promega) in AB. Supernatants were collected and completely dried under speed vacuum. Samples were then desalted with a C18 microspin column (Harvard Apparatus) according to the manufacturer’s instructions, completely dried under speed vacuum and stored at −20°C.

#### Liquid chromatography-electrospray ionization tandem mass spectrometry (LC-ESI-MSMS)

Samples were diluted in 20 µL loading buffer (5% acetonitrile [CH_3_CN], 0.1% formic acid [FA]) and 2 µL was injected into the column. LC-ESI-MS/MS was performed either on a Q-Exactive Plus Hybrid Quadrupole-Orbitrap Mass Spectrometer (Thermo Fisher Scientific) equipped with an Easy nLC 1000 liquid chromatography system (Thermo Fisher Scientific) or an Orbitrap Fusion Lumos Tribrid mass Spectrometer (Thermo Fisher Scientific) equipped with an Easy nLC 1200 liquid chromatography system (Thermo Fisher Scientific). Peptides were trapped on an Acclaim pepmap100, 3 µm C18, 75 µm × 20 mm nano trap-column (Thermo Fisher Scientific) and separated on a 75 µm × 250 mm (Q-Exactive) or 500 mm (Orbitrap Fusion Lumos), 2 µm C18, 100 Å Easy-Spray column (Thermo Fisher Scientific). The analytical separation used a gradient of H_2_O/0.1% FA (solvent A) and CH_3_CN/0.1% FA (solvent B). The gradient was run as follows: 0–5 min 95% A and 5% B, then to 65% A and 35% B for 60 min, then to 10% A and 90% B for 10 min, and finally for 15 min at 10% A and 90% B. Flow rate was 250 nL/min for a total run time of 90 min.

Data-dependant analysis (DDA) was performed on the Q-Exactive Plus with MS1 full scan at a resolution of 70,000 full width at half maximum (FWHM) followed by MS2 scans on up to 15 selected precursors. MS1 was performed with an AGC target of 3 × 10^6^, a maximum injection time of 100 ms and a scan range from 400 to 2,000 *m/z*. MS2 was performed at a resolution of 17,500 FWHM with an automatic gain control (AGC) target at 1 × 10^5^ and a maximum injection time of 50 ms. Isolation window was set at 1.6 *m/z* and 27% normalized collision energy was used for higher-energy collisional dissociation (HCD). DDA was performed on the Orbitrap Fusion Lumos with MS1 full scan at a resolution of 120,000 FWHM followed by as many subsequent MS2 scans on selected precursors as possible within a 3-s maximum cycle time. MS1 was performed in the Orbitrap with an AGC target of 4 × 10^5^, a maximum injection time of 50 ms and a scan range from 400 to 2,000 *m/z*. MS2 was performed in the Ion Trap with a rapid scan rate, an AGC target of 1 × 10^4^ and a maximum injection time of 35 ms. Isolation window was set at 1.2 *m/z* and 30% normalized collision energy was used for HCD.

#### Database searches

Peak lists (MGF file format) were generated from raw data using the MS Convert conversion tool from ProteoWizard. The peak list files were searched against the PlasmoDB_*P.berghei* ANKA database (PlasmoDB.org [46], release 38, 5076 entries) combined with an in-house database of common contaminants using Mascot (Matrix Science, London, UK; version 2.5.1). Trypsin was selected as the enzyme, with one potential missed cleavage. Precursor ion tolerance was set to 10 ppm and fragment ion tolerance to 0.02 Da for Q-Exactive Plus data and to 0.6 for Lumos data. Variable amino acid modifications were oxidized methionine and deamination (Asn and Gln) as well as phosphorylated serine, threonine, and tyrosine. Fixed amino acid modification was carbamidomethyl cysteine. The Mascot search was validated using Scaffold 4.8.4 (Proteome Software) with 1% of protein false discovery rate (FDR) and at least two unique peptides per protein with a 0.1% peptide FDR.

#### Principal component analyses

Proteins identified in the WT control from reference 13 were removed from the analysis. For PCA, IPs of FBXO1-HA performed under the same experimental conditions (13) were used as an additional control. Quantitative value (normalized total spectra) was calculated by Scaffold. Missing values were imputed with the minimum fixed value of 0.5 and principal component analysis was computed and plotted using R.

### Proteomic analyses

Proteomic analyses were performed as previously described in reference 22.

#### Sample preparation for proteomic characterization of gametocytes

Cell lysis of two independent biological replicates was performed in 100 µL of 2% SDS, 25 mM NaCl, 50 mM Tris (pH 7.4), 2.5 mM EDTA, and 20 mM TCEP supplemented with 1× Halt protease and phosphatase inhibitor. Samples were vortexed and then heated at 95°C for 10 min with 400 rpm mixing with a thermomixer. DNA was sheared with four sonication pulses of 10 s each at 50% power. Samples were centrifuged for 30 min at 17,000 × *g* and supernatants were collected. Protein concentration of each sample was estimated using the Pierce BCA protein assay kit (Thermo Fisher) following the manufacturer’s instructions. Samples were then diluted with 200 µL of 50 mM Tris (pH 7.4) and incubated with 48 µL of iodoacetamide 0.5 M for 1 h at room temperature. Proteins were digested based on the FASP method using Amicon Ultra-4, 30 kDa as centrifugal filter units (Millipore). Trypsin (Promega) was added at a 1:80 enzyme:protein ratio and digestion was performed overnight at room temperature. The resulting peptide samples were desalted with Pierce Peptide Deslting Spin Column (Thermo Fisher Scientific) according to the manufacturer’s instruction; peptide concentration was determined using a colorimetric peptide assay (Thermo Fisher Scientific) then completely dried under speed vacuum.

#### ESI-LC-MSMS

Duplicate samples were dissolved at 1 µg/µL with loading buffer (5% CH3CN, 0.1% FA). Biognosys iRT peptides were added to each sample and 2 µg of peptides were injected into the column. LC-ESI-MS/MS was performed on an Orbitrap Fusion Lumos Tribrid mass spectrometer (Thermo Fisher Scientific) equipped with an Easy nLC1200 liquid chromatography system (Thermo Fisher Scientific). Peptides were trapped on a Acclaim pepmap100, C18, 3 µm, 75 µm × 20 mm nano trap-column (Thermo Fisher Scientific) and separated on a 75 µm × 500 mm, C18 ReproSil-Pur (Dr. Maisch GmBH), 1.9 µm, 100 Å, home-made column. The analytical separation was run for 135 min using a gradient of H_2_O/FA 99.9%/0.1% (solvent A) and CH_3_CN/H_2_O/FA 80.0%/19.9%/0.1% (solvent B). The gradient was run from 8% B to 28% B in 110 min, then to 42% B in 25 min, then to 95%B in 5 min with a final stay of 20 min at 95% B. Flow rate was 250 nL/min and total run time was 160 min. DIA was performed with MS1 full scan at a resolution of 60,000 (FWHM) followed by 30 DIA MS2 scan with fixed windows. MS1 was performed in the Orbitrap with an AGC target of 1 × 10^6^, a maximum injection time of 50 ms and a scan range from 400 to 1,240 *m/z*. DIA MS2 was performed in the Orbitrap using higher-energy collisional dissociation (HCD) at 30%. Isolation windows were set to 28 *m/z* with an AGC target of 1 × 10^6^ and a maximum injection time of 54 ms.

#### Data analysis

DIA raw files were loaded separately on Spectronaut v.15 (Biognosys) and analyzed by directDIA using default settings (two .SNE file have been generated). Briefly, data were searched against *Plasmodium berghei* ANKA database (PlasmoDB.org, release 49, 5,076 entries). Trypsin was selected as the enzyme, with two potential missed cleavages. Variable amino acid modifications were oxidized methionine and GlyGly (GG) derivatization of lysine (+114 Da) (K). Fixed amino acid modification was carbamidomethyl cysteine. Both peptide precursor and protein FDR were controlled at 1% (*Q* value < 0.01). Single Hit Proteins were excluded for Super Natant samples. For quantitation, top three precursor areas per peptide were used, “only protein group specific” was selected as proteotypicity filter and normalization was set to “automatic.” A paired *t* test was applied for differential abundance testing. The following parameters were used: Quantity at the MS2 level, quantity type = area, data filtering by Qvalue, and cross-run normalization selected. Proteins and peptides were considered to have significantly changed in abundance with a *Q* value ≤0.05 and an absolute fold change FC ≥|1.5| (log_2_FC ≥|0.58|).

### Enrichment analysis

Gene ontology (GO) term enrichment analysis, as well as associated plots, were performed with ClusterProfiler (47, 48) R package, using the EnrichGO function as previously described in reference (22). Enrichment tests were calculated for GO terms, based on hypergeometric distribution. *P* value cutoff was set to 0.05. The gofilter function was used prior to cnetplot drawing, to filter results at specific levels.

### Softwares

ImageJ 1.53, and LAS X (Leica version 3.5.7.23225) were used for image analysis. Excel (v1108) and GraphPad Prism 9 (9.5.1) were used for data and statistical analysis. Bio-Rad Image Lab (6.1) was used for western blot analysis. The R package was used for proteomic data analysis and representation. ClusterProfiler (3.8) was used for Gene Ontologies term enrichment analysis. Spectronaut v.15 (Biognosys) was used for DIA analysis. Mascot (Matrix Science, London, UK; version 2.5.1) was used for the peak list files searches against the PlasmoDB_P.berghei ANKA database (PlasmoDB.org, release 38). ZEN 2.6 (Zeiss) and LAS X (Leica version 3.5.7.23225) were used for image acquisition.

## Data Availability

All data needed to evaluate the conclusions in the paper are present in the paper and/or the supplemental materials. The WIG1 proteomic data have been deposited to the ProteomeXchange Consortium via the PRIDE partner repository (http://proteomecentral.proteomexchange.org) with the data set identifier PXD035557.

## SUPPLEMENTAL MATERIAL

Figure S1 mbio.01672-24-s0001.pdf SLI plasmid and PbCUL4-HA results.

Figure S2 mbio.01672-24-s0002.pdf Genetic modification strategy for WIG1 gene disruption in *P. berghei* and genotyping data.

Figure S3 mbio.01672-24-s0003.pdf WIG1 disruption.

Legends mbio.01672-24-s0004.docx Supplemental figure and table legends.

Table S1 mbio.01672-24-s0005.xlsx Proteins detected in CUL4-HA immunoprecipitates from *P. falciparum* schizonts.

Table S2 mbio.01672-24-s0006.xlsx Proteins detected in CUL4-HA, DDB1-HA and WIG1-HA immunoprecipitates from *P. berghei* gametocytes.

Table S3 mbio.01672-24-s0007.xlsx Proteome comparison of WT and WIG1-GD gametocytes four minutes post-activation by XA.

Table S4 mbio.01672-24-s0008.xlsx Oligonucleotides used in this study.

Table S5 mbio.01672-24-s0009.xlsx Main reagents used or generated in this study.

ASM does not own the copyrights to Supplemental Material that may be linked to, or accessed through, an article. The authors have granted ASM a non-exclusive, world-wide license to publish the Supplemental Material files. Please contact the corresponding author directly for reuse.
