# Supplementary figures and images for: A multistage Plasmodium CRL4WIG1 ubiquitin ligase is critical for the formation of functional microtubule organization centers in microgametocytes
Source: mBio. 2024 Aug 29;15(10):e01672-24. doi: 10.1128/mbio.01672-24 (PMC11481892; doi:10.1128/mbio.01672-24)

Figure S1

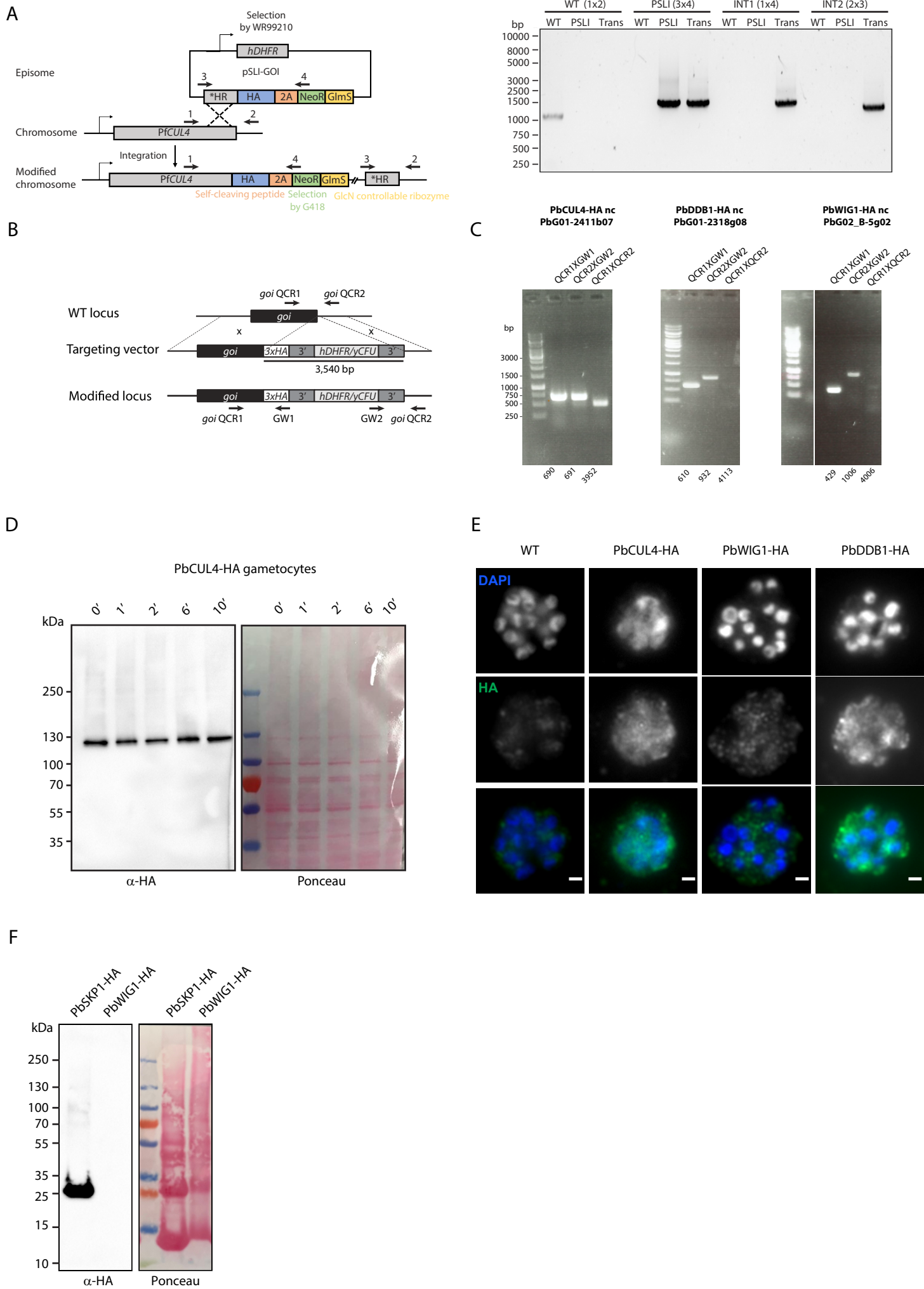

Supplement: Figure S1 — SLI plasmid and PbCUL4-HA results. [file mbio.01672-24-s0001.pdf]

Figure S2

A

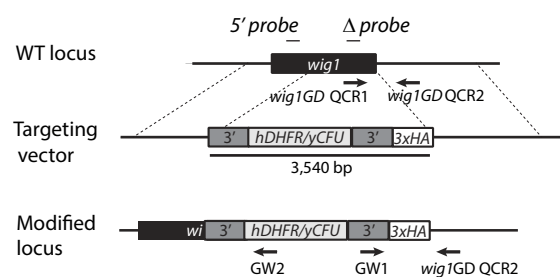

B

WIG1-GD clonal  
PbG02\_B-5g02

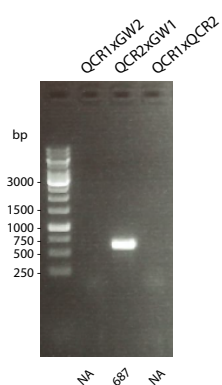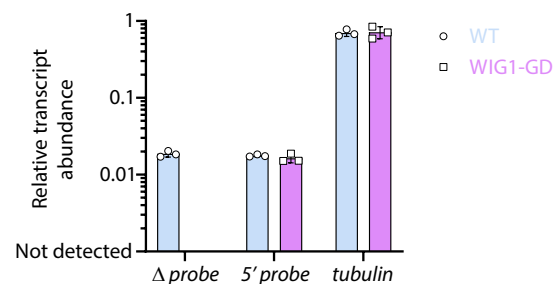

C

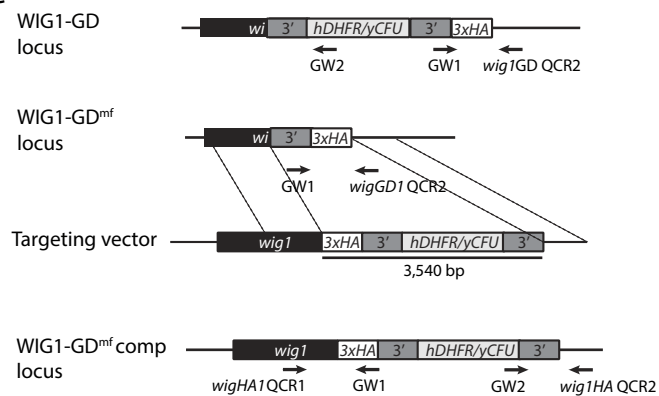

WIG1-GD<sup>mf</sup> comp

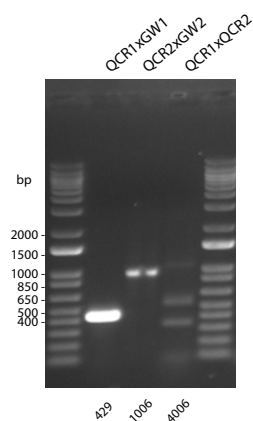

Supplement: Figure S2 — Genetic modification strategy for WIG1 gene disruption in P. berghei and genotyping data. [file mbio.01672-24-s0002.pdf]

Figure S3

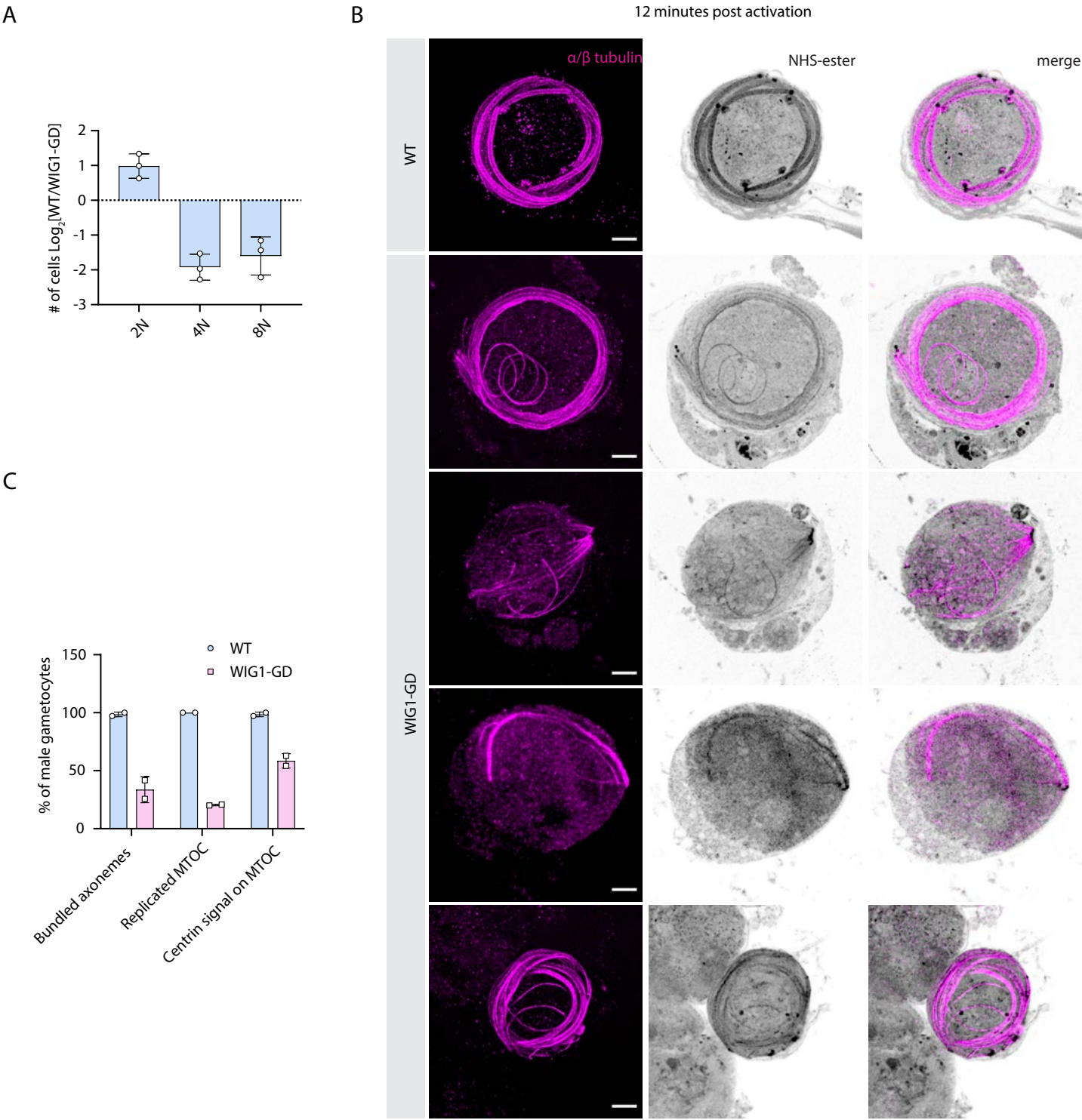

Supplement: Figure S3 — WIG1 disruption. [file mbio.01672-24-s0003.pdf]
