# Supplementary material for: A multistage Plasmodium CRL4WIG1 ubiquitin ligase is critical for the formation of functional microtubule organization centers in microgametocytes
Source: mBio. 2024 Aug 29;15(10):e01672-24. doi: 10.1128/mbio.01672-24 (PMC11481892; doi:10.1128/mbio.01672-24)
Supplement: Legends — Supplemental figure and table legends. [file mbio.01672-24-s0004.docx]

**Supplementary material**

**Supplementary figure legends**

**Figure S1. A.** Diagrammatic representation of the SLI plasmid and the mechanism of single cross-over homologous recombination resulting from spontaneous DNA modification around the targeted locus. Numbered arrows represent primers which are used in the subsequent PCR mediated validation. Representative PCR analysis using specific primer pairs designed to amplify the SLI plasmid (3 + 4), the wild type locus (1 + 2), and the modified locus (INT1: 1 + 4, INT2: 3 + 2) using template DNA of the SLI plasmid or that derived from wild type or electroporated and selected *P. falciparum*. **B.** Western blot analysis of PfCUL4-HA expression in *P. falciparum* lysates collected at different time points during an intra erythrocytic cycle. **C and D.** Genetic modification strategy for HA tagging in *P. berghei* and genotyping data (C). Oligonucleotides used for PCR genotyping are indicated and agarose gels for corresponding PCR products from genotyping reactions are shown (D). The same marker size was used for all genotyping gels. PCR product sizes are indicated below each lane; NA: not amplifiable. In the line names nc is for non-clonal lines, the ID below is the PlasmoGEM vector from which the construct was derived. **E.** Western blot analysis of PbCUL4-HA gametocyte lysates over the course of gametogenesis as shown in Fig. 1. The Ponceau staining serves as a loading control. The blot is representative of 2 independent replicates. **F.** Western blot analysis of a gametocyte lysate from the line expressing endogenously HA-tagged WIG1 does not allow to detect the fusion protein as shown in Fig. 1. The blot is representative of 2 independent replicates. A lane loaded with a PbSKP1-HA gametocyte lysate serves as a positive control. A Ponceau staining serving as a loading control. **G.** Localisation of PbCUL4-HA, PbDDB1-HA and WIG1-HA (all green), by widefield immunofluorescence in segmenting schizonts. A WT line is shown as a control. DNA is stained with DAPI (blue). Scale bars = 1 µm.

**Figure S2. A.** Genetic modification strategy for *WIG1* gene disruption in *P. berghei* and genotyping data. Oligonucleotides used for PCR genotyping are indicated and agarose gels for corresponding PCR products from genotyping reactions are shown. The same marker size was used for all genotyping gels. PCR product sizes are indicated below each lane; NA: not amplifiable. The ID below the line name is the PlasmoGEM vector from which the construct was derived. **B.** qRT-PCR showing normalised expression of *WIG1* transcripts in WT and WIG1-GD gametocytes. Shown is mean ± standard deviation; n = 3 independent experiments. **C.** Genetic modification strategy to cis-complement the *WIG1* gene disruption in *P. berghei* and genotyping data. Oligonucleotides used for PCR genotyping are indicated and agarose gels for corresponding PCR products from genotyping reactions are shown. PCR product sizes are indicated below each lane.

**Figure S3. A.** Effect of WIG1 disruption 15 minutes after XA activation on microgametocytes ploidy as assessed by flow cytometry on vibrant violet stained cells (n = 3 independent biological replicates). **B.** Gallery of maximum projections showing the effect of WIG1 disruption on microgametogenesis as observed by U-ExM 12 minutes after activation by XA. α/β-Tubulin: magenta; amine reactive groups/NHS-ester: shades of grey. Scale bars = 5 µm. **C.** Quantification of the main phenotypes shown in Figure 3D and 3E in 35 cells from independent biological duplicates.

**Supplementary tables**

**Table S1.** Proteins detected in CUL4-HA immunoprecipitates from *P. falciparum* schizonts

**Table S2.** Proteins detected in CUL4-HA, DDB1-HA and WIG1-HA immunoprecipitates from *P. berghei* gametocytes

**Table S3.** Proteome comparison of WT and WIG1-GD gametocytes four minutes post-activation by XA

**Table S4.** Oligonucleotides used in this study

**Table S5.** Main reagents used or generated in this study
